# Evolution and inheritance of animal mitochondrial DNA: rules and exceptions

**DOI:** 10.1186/s40709-017-0060-4

**Published:** 2017-01-31

**Authors:** Emmanuel D. Ladoukakis, Eleftherios Zouros

**Affiliations:** Department of Biology, University of Crete, Voutes University Campus, 70013 Iraklio, Greece

**Keywords:** mtDNA, Evolution, Inheritance, Recombination, Heteroplasmy

## Abstract

Mitochondrial DNA (mtDNA) has been studied intensely for “its own” merit. Its role for the function of the cell and the organism remains a fertile field, its origin and evolution is an indispensable part of the evolution of life and its interaction with the nuclear DNA is among the most important cases of genome synergism and co-evolution. Also, mtDNA was proven one of the most useful tools in population genetics and molecular phylogenetics. In this article we focus on animal mtDNA and discuss briefly how our views about its structure, function and transmission have changed, how these changes affect the information we have accumulated through its use in the fields of phylogeny and population structure and what are the most important questions that remain open for future research.

## Background

Mitochondria are organelles, remnants of ancestral bacterial endosymbionts, found in nearly all eukaryotic cells [1]. Mitochondria, along with plastids in plants, are the only cytoplasmic organelles in the eukaryotic cell that carry genetic elements. In the last release of organelle section of the genome database of NCBI (June 2016) there were 6955 complete mtDNA sequences of which 6253 (~90%) belong to animals. Of the latter 4024 (64%) belong to metazoans. This bias for metazoan mtDNA does not allow for a complete picture of mtDNA variation in the entire eukaryotic world.

This review is, also, restricted to animal mtDNA, which is a relatively conserved molecule [2]. In most animals the mtDNA is a short, circular molecule that contains about 13 intronless, protein-coding genes, all of which are involved in oxidative phosphorylation (OXPHOS), a process also known as aerobic respiration. Animal mtDNA also contains two rRNA genes and 22 or 23 tRNA genes, which are part of the translational machinery of the mitochondrion. With few exceptions, non-coding regions in the mtDNA molecule are few and short, apart from the region that contains the regulatory elements for replication and transcription. This region is known as large non-coding region and its length and position within the molecule vary greatly among species. The prevailing view is that animal mtDNA is maternally transmitted, non-recombining and with elevated mutation rate compared to nuclear DNA.

As noted, this general description of animal mtDNA cannot be extended to all eukaryotes. If we compare animals, fungi, protists and plants, we will find that their mtDNAs differ drastically in all major characteristics. MtDNA is an extremely variable genome, perhaps more variable than the nuclear genome. The variability is not surprising, given the 2 billion years of mtDNA evolution [3, 4]. Even within animals, the variation is much more than the traditional view of animal mtDNA conservatism would imply. In the following paragraphs we list and discuss shortly some of the most important variations we know in the metazoan mtDNA.

### Functions and uses of mtDNA

Mitochondria have been characterized as the powerhouses of the cell, because their most basic function is OXPHOS. The coding and synthesis of proteins that are integral parts of enzymatic complexes that catalyze OXPHOS, remains the most important role of mtDNA, and it is the function of mtDNA we find in all eukaryotes. However, mitochondria participate in several other, important cellular processes, such as apoptosis [5], ageing [6], signalling [7], metabolic homeostasis and biosynthesis of important macromolecules such as lipids and heme [8, 9]. It is only recently that we have become aware of the multiplicity of functions that mtDNA coded proteins may exhibit, beyond their participation in OXPHOS [10]. Examples include NAD2 which interacts with Src, a tyrosine kinase which is critical for controlling important cellular functions [11]. The males of several freshwater mussels carry a COX2 gene with a long extension of approximately 185 amino acid residues, which is supposed to play a role in the biparental transmission of mtDNA, known as doubly uniparental inheritance—DUI mechanism [10, 12] (we discuss DUI below). Inversely, there are mtDNA-encoded peptides that do not participate in OXPHOS, and are suspected to play important roles in other cellular functions. For instance, a small peptide that is encoded in the 16S RNA gene of human is suspected to act as an oncogene [13]. Another example is a small peptide (of only 14 aa) that is encoded in the control region of the male mtDNA of various bivalves with DUI, for which it is suggested that it plays a role in this exceptional mode of mtDNA transmission [14, 15]. Other mtDNA encoded peptides have been found in several animal species, but their functional role remains uncertain [10].

Mutations in mtDNA genes have been implicated in several severe genetic diseases in humans. These mutations are either point substitutions or deletions of varying length [16]. There is no general pattern in the causes of mitochondrial diseases, but it seems that they result from a heteroplasmic state in which the defected mtDNA co-occurs with normal mtDNA. Since there is no meiotic control in mtDNA replication, the defected molecule can increase its frequency in the cell either stochastically or because its smaller size allows for a faster replication rate. It has been suggested that the diseased phenotype appears when the frequency of the defected mtDNA surpasses a threshold, which varies from mutation to mutation and from tissue to tissue [17]. Mitochondrial diseases can be extremely complex and their treatment is difficult. Their importance has led to the development of the field of mitochondrial medicine. A well known success of mitochondrial medicine is the, so called, “three parent embryos” method, which consists in the dilution of the defected maternal mitochondria with mitochondria from a third person (thus, the embryo has two nuclear DNA parents and a different mtDNA parent) [18].

It would be no exaggeration to say that mtDNA is responsible for the explosion of studies in population genetics and molecular phylogenetics in the last quarter of past century. The first studies of animal mtDNA revealed that it possesses characteristics that make it an ideal genetic marker [19]. Maternal inheritance and absence of recombination are very desirable properties for a tool in the reconstruction of phylogenetic histories because it allows tracing each lineage as a single evolutionary history. Further, the existence of conserved and less conserved regions within the same molecule and the elevated mutation rate relative to the nuclear DNA make mtDNA suitable for comparisons both among individuals from the same population and among distantly related species.

These intrinsic properties of mtDNA as a tool for population genetic studies were complemented with its rather inexpensive use. The alternation of variable and conserved regions on the same molecule allowed the design of universal primers which could amplify pieces of the mtDNA of practically any species, without previous knowledge about the species’ mtDNA. Multiple copies of mtDNA within each cell made the amplification of the mtDNA easier than parts of the nuclear DNA. Homoplasmy of mtDNA made feasible the direct sequencing of the PCR product, unlike nuclear genes where the maternal and paternal alleles need to be separated before sequencing. Both the intrinsic properties and the technical ease made the mtDNA perhaps the most popular genetic marker before the advent of large scale sequencing. But these celebrated natural properties of mtDNA proved to be rules with exceptions. Ruther than being a nuisance, these exceptions helped us to get a deeper insight into the molecule’s evolution and function, as we will show in the subsequent paragraphs.

### Size, shape and gene content variation

Animal mtDNA is normally a circular, compact molecule about 17 Kb with little variation in size, containing 13 protein coding genes, 2 rRNA and 22 tRNA genes. This pattern is conserved among bilaterians, with few exceptions. However, in non-bilaterians there is high variation in size, shape, gene content and genetic code of mtDNA (for a comprehensive review of the variation of mtDNA among non-bilaterian animals, see [20]) . The smallest metazoan mtDNA is that of the Ctenophore *Mnemiopsis leidyi*, which is just above 10 Kb [21], and the largest of the bivalve *Anadara sativa,* with a length over 48 Kb. Within all eukaryotes, mtDNA varies greatly in size and gene content. The mtDNA of the cucumber (*Cucumis sativus*) is 1556 Kb and carries 65 genes, while that of the fungus *Cryphonectria parasitica* is only 1.3 Kb and carries one gene. Some plants have smaller mtDNAs than that of the cucumber but carry more genes. Such is the mtDNA of the pepper *Capsicum annuum* which is 511 Kb and carries 221 genes. The variation in size becomes even larger if we take into account organelles, such as mitosomes and hydrogenosomes, which derive from the mitochondrion but lack DNA and maintain only a double membrane [22, 23].

The mtDNA of some animals is not a single circular molecule, but occurs as two or more circular or linear “chromosomes” [24–28]. The number of these chromosomes varies from two (e.g. in *Liposcelis bostrychophila* [29]) to as high as 20 in the human body louse, *Pediculus humanus* [27]. Similarly, an examination of the NCBI organelle genome database reveals that the GC content of animal mtDNA varies from 11 to 57%.

### Maternal transmission and heteroplasmy

There are several exceptions to the rule that mtDNA is maternally transmitted. In some plants (e.g. most conifers [30] and cucumber [31]) mtDNA is paternally transmitted through pollen. In animals, a well known and idiomorphic case of paternal transmission is that of doubly uniparental inheritance (DUI) that is found in several species of molluscan bivalves [32, 33]. In this peculiar mode of inheritance, females transmit their mtDNA to both male and female offspring and males transmit their mtDNA only to male offspring. The result is the co-occurrence in the same species of two independently evolving mtDNA lineages, one that is transmitted through the eggs and another through the sperm. Consequently, females are basically homoplasmic for the maternal mtDNA (but may contain low amounts of paternal mtDNA) and produce eggs with only the maternal mtDNA. Males are heteroplasmic for both the maternal and the paternal mtDNA but produce sperm that contains only the latter.

The mechanisms that ensure maternal transmission of mtDNA vary in different organisms [34]. For example, in mammals sperm mitochondria are ubiquitinated and subsequently destroyed [35]; in *Drosophila*, mitochondria are destroyed during spermatid formation [36]; in *Oryzias latipes*, the mitochondria of the sperm are actively destroyed [37] and in *Caenorhabditis elegans* the sperm’s mitochondria are destroyed through autophagy [38, 39]. Maternal inheritance of mtDNA leads to homoplasmic individuals, i.e. individuals that have a single type of mtDNA. Homoplasmy is further reinforced by pre- and a post- fertilization bottlenecks [40]. The pre-fertilization bottleneck occurs during oogenesis, where the number of mitochondria is severely reduced in the germ line, before maturation of the oocyte. The post-fertilization bottleneck occurs between the zygote formation and the blastocyst embryonic stages, during which there is intense cell division but suppression of mitochondrial proliferation, a mechanism that leads to a reduced number of mitochondria per cell.

The variation of the mechanisms that have been evolved to ensure maternal transmission of mtDNA and the ubiquity of this transmission mode among organisms indicate that there must be a strong evolutionary reason for the maintenance of maternal transmission. The most prominent hypothesis for maternal transmission of mtDNA is that uniparental (maternal in the case of metazoans) transmission of mtDNA prevents the spread of selfish (fast replicating) mutations in the population [41–43]. This hypothesis is supported by experiments in yeast, where yeast cells with a small, defective mtDNA molecule replicate faster than the normal mtDNA, but produce smaller colonies (petit) relative to the cells with normal mtDNA [44]. Recently, a second hypothesis—not necessarily mutually exclusive to the above—suggests that maternal transmission has evolved to prevent heteroplasmy [45]. Heteroplasmy has been involved in mitochondrial diseases [46]. Experimental evidence from mice have shown that heteroplasmy can cause severe physiological, cognitive and behavioral problems [47]. In *Drosophila* though, there are reports that heteroplasmy is adaptive. When both molecules contained a deleterious mutation, different in each molecule, the presence of both molecules in the same individual cancels out the deleterious effects caused when each molecule occurs alone [48].

Given the strictness of the mechanisms that prevent paternal leakage of mtDNA, heteroplasmy should be extremely rare in nature. However, more and more publications reported heteroplasmy in natural populations in several species such as anchovy [49], *Drosophila* [50], mice [51], oniscid crustaceans [52], frogs [53] and humans [54, 55]. Despite the increased number of reports of heteroplasmy, the proportion of species in which heteroplasmy has been observed remains low, compared to the large number of species that sequences for their mtDNA have been deposited in GenBank. One possibility is that heteroplasmy is common but it cannot be easily detected with the techniques used. The most commonly used technique involves PCR-amplification of a specific segment of the mtDNA molecule and subsequent sequencing, either directly or after cloning. If the targeted mtDNA pool contains a predominant molecule and some other types in low frequencies, the traditional Sanger sequencing will not reveal the presence of the rare molecules. Only the design of specific primers for the rare molecules could detect them when using direct sequencing. Alternatively, a large number of clones from the PCR product need to be sequenced, or next generation sequencing (NGS) should be applied, to reveal the presence of rare molecules. The accuracy of next generation sequencing allows not only to detect heteroplasmy when it is present [e.g. 54], but also to eliminate the possibility of heteroplasmy if no evidence for it is obtained. In a recent study, paternal transmission was excluded from four human parents-offspring trios [56].

Heteroplasmy may result through different routes, not mutually exclusive. First, the egg may be heteroplasmic, containing two or more different types; this is the case of mother-inherited heteroplasmy. Second, somatic mutations may occur during mtDNA replication in somatic cells. Given the tremendous amount of mtDNA copies per individual (a diploid organism may contain billions of cells and each cell contains two copies of nuclear genes but hundreds or thousands of copies of mtDNA) and the elevated mutation rate of mtDNA in animals, each individual contains unavoidably many mutated forms of the mtDNA which it inherited from its mother. Finally, leakage of paternal mtDNA can be a significant source of heteroplasmy. The mitochondria of the sperm may escape destruction in the egg during fertilization so that the embryo will be heteroplasmic for a maternal and a paternal mtDNA molecule.

Some authors suggested that leakage of paternal mtDNA occurs accidentally because the mechanism that supervises uniparental transmission is not infallible [57]. The chance of paternal leakage in offspring will be higher when the genetic distance between the two parents is high. The reason for this is failure of the egg-sperm mitochondrial recognition mechanism. Basically, the mechanism consists of a factor that is coded by the maternal nuclear genome and occurs in the eggs, and of a signal that is coded by the paternal genome and occurs in the outer surface of sperm mitochondria. When the latter enter the egg their signal is recognized by the factor and destruction of sperm mitochondria ensues. A sequel of this hypothesis is that the mechanism will be less efficient the more divergent are the maternal and paternal species. The hypothesis suggests that heteroplasmy will be more common in progeny from heterospecific than from homospecific crosses. Evidence from *Drosophila* and frogs supports this hypothesis [53, 58, 59], but further support is needed. Other authors have suggested that paternal leakage might be under the control of natural selection [59].

The overall evidence suggests that heteroplasmy is common to the point that strictly maternal inheritance cannot be held as a rule. This would be true if we consider maternal inheritance as a qualitative character or, alternatively, if we consider paternal leakage as a presence/absence trait. But it might be more useful to consider maternal transmission as a quantitative characteristic [60]. If we do so, then the answer is that in animals an overwhelming amount of mtDNA is maternally transmitted and that paternal leakage is restricted to very low amounts.

### Recombination

Recombination in plants and fungi mtDNA has been reported in the early 80s of past century [61, 62], but animal mtDNA was considered for decades as a non-recombining genome [63]. The view that there is no recombination in the animal mtDNA was based on the assumption of homoplasmy, itself a result of the assumption of strict maternal transmission. The view was supported by the persistent lack of evidence for recombination. But experiments showed that animal mitochondria contain the enzymatic apparatus for recombination [64]. The first direct evidence for recombination was obtained by Ladoukakis and Zouros [65] in mussels. This was followed by a long list of mtDNA recombination in other organisms, including human [66] and *Drosophila* [67], using either direct sequencing or utilizing data deposited in GenBank [68–71]. Like heteroplasmy, the detection of recombination is not easy given the rarity of recombinant molecules in an individual. However, next generation sequencing (NGS) techniques promise to be a powerful tool for the detection of recombinants, given their ability to detect molecules in a DNA pool that occur in very low amounts. But NGS can also produce artificial recombinants (chimeric sequences) in a low frequency [72]. Evidence for real recombination requires, therefore, that the detected recombinants exceed the error threshold of the used technology. Using these sensitive techniques Kraytsberg et al. [66] detected recombination in human mtDNA and Hagstrom et al. [73] were able to exclude mtDNA recombination in mice.

The evolutionary consequences of recombination of mtDNA are far reaching. Non recombining genomes accumulate deleterious mutations much faster than recombining ones through the mechanism known as Muller’s ratchet [74, 75]. ΜtDNA, which originated from symbiosis of an a-proteobacterium with an archaeobacterium [76] about 2 billion years ago [3], should have collapsed under the burden of accumulated deleterious mutations [77]. The elevated mutation rate and the low effective population size of animal mtDNA, which is estimated to be one quarter of that of nuclear autosomes [78] makes the mtDNA more prone to accumulation of deleterious mutations. That the mtDNA molecule has not collapsed may be due to recombination. It is known that even a very low recombination rate would be sufficient to cancel Muller’s ratchet [79, 80] and rescue mtDNA from deleterious mutation meltdown.

The presence of mtDNA recombination may have had an undesirable effect on its use as a genetic marker. Simulations have shown that a phylogenetic tree reconstructed with mtDNA sequences that were allowed to recombine had, in comparison with a tree in which mtDNA recombination was not allowed, longer terminal branches, larger total branch lengths and shorter times to the most recent common ancestor [81]. However, more research is needed to appreciate the effects of mtDNA recombination on phylogeny reconstruction, particularly with the extensive use of Bayesian methods on phylogeny.

## Concluding remarks

The history of our understanding of animal mtDNA is itself a lesson of how we gain knowledge of the complexity in biology. In the early 80s of past century animal mtDNA was a very accommodating molecule, God’s gift to those interested in phylogeny, taxonomy and population genetics: small, conservative in gene arrangement, with slow and fast diverging segments, uniparentally inherited, homoplasmic, non-recombining, with one function (the OXPHOS), easy to study. The result was an explosion of our knowledge of the tree of life from its roots and big branches to tiny bifurcations at the top. Gradually the molecule presented us its true face. No “rule” about it remained unbroken. The size of 18 Kb is valid as a mean, but there is large variation around it. Maternal inheritance, and therefore homoplasmy, can be bypassed in many ways, and recombination occurs when conditions allow it to occur, i.e., fusion of mitochondria with different mtDNAs. Perhaps more important than the above, animal mtDNA proved to be anything but a single-purpose molecule. It is involved in male fertility, in the action of muscles and neurons, in ageing and in sex inheritance. Its interactions with the nuclear genome remain largely unexplored, but are so serious that the mtDNA has become an important factor in cloning of cells and embryos. A whole field of medical science has developed around human mtDNA. Nuclear DNA and mtDNA have been co-evolving synergistically in ensuring the well functioning of the organism that carries them, and antagonistically in their race for long term existence. In this antagonism the nucleus is under pressure to impose and maintain a uniparental inheritance of mtDNA and to increase its independence from mtDNA by “stealing” functional genes from it. The mtDNA, on the other hand, is under constant pressure to evade uniparental inheritance, avoid mutational meltdown, and increase its indispensability for the organism by incorporating information that is necessary for the organism’s function. Perhaps this complexity is expected form a molecule that started the most fundamental symbiosis for the eukaryotic world since two billion years ago. Most of the information we gained about the tree of life through the use of mtDNA remains valid. But this role of the mtDNA fades away rapidly as our reading of DNA sequences becomes more powerful (and efficient). This being so, the future of mtDNA research must be in its role in the function of the organism and its value as a tool in the study of major evolutionary novelties in the history of life.

## References

[1] Gray MW, Burger G, Lang BF (1999). Mitochondrial evolution. Science.

[2] Gissi C, Iannelli F, Pesole G (2008). Evolution of the mitochondrial genome of Metazoa as exemplified by comparison of congeneric species. Heredity.

[3] Lang BF, Gray MW, Burger G (1999). Mitochondrial genome evolution and the origin of eukaryotes. Annu Rev Genet.

[4] Lane N, Martin W (2010). The energetics of genome complexity. Nature.

[5] Sinha K, Das J, Pal PB, Sil PC (2013). Oxidative stress: the mitochondria-dependent and mitochondria-independent pathways of apoptosis. Arch Toxicol.

[6] Bratic A, Larsson NG (2013). The role of mitochondria in aging. J Clin Investig.

[7] Chandel NS (2015). Evolution of mitochondria as signaling organelles. Cell Metab.

[8] Cheng Z, Ristow M (2013). Mitochondria and metabolic homeostasis. Antioxid Redox Signal.

[9] Ahn CS, Metallo CM (2015). Mitochondria as biosynthetic factories for cancer proliferation. Cancer Metab.

[10] Breton S, Milani L, Ghiselli F, Guerra D, Stewart DT, Passamonti M (2014). A resourceful genome: updating the functional repertoire and evolutionary role of animal mitochondrial DNAs. Trends Genet.

[11] Gingrich JR, Pelkey KA, Fam SR, Huang Y, Petralia RS, Wenthold RJ (2004). Unique domain anchoring of Src to synaptic NMDA receptors via the mitochondrial protein NADH dehydrogenase subunit 2. Proc Natl Acad Sci USA.

[12] Chakrabarti R, Walker JM, Chapman EG, Shepardson SP, Trdan RJ, Curole JP (2007). Reproductive function for a C-terminus extended, male-transmitted cytochrome c oxidase subunit II protein expressed in both spermatozoa and eggs. FEBS Lett.

[13] Maximov V, Martynenko A, Hunsmann G, Tarantul V (2002). Mitochondrial 16S rRNA gene encodes a functional peptide, a potential drug for Alzheimer’s disease and target for cancer therapy. Med Hypotheses.

[14] Breton S, Ghiselli F, Passamonti M, Milani L, Stewart DT, Hoeh WR (2011). Evidence for a fourteenth mtDNA-encoded protein in the female-transmitted mtDNA of marine Mussels (Bivalvia: Mytilidae). PLoS ONE.

[15] Milani L, Ghiselli F, Guerra D, Breton S, Passamonti M (2013). A comparative analysis of mitochondrial ORFans: new clues on their origin and role in species with doubly uniparental inheritance of mitochondria. Genome Biol Evol.

[16] Lightowlers RN, Taylor RW, Turnbull DM (2015). Mutations causing mitochondrial disease: what is new and what challenges remain?. Science.

[17] Lightowlers RN, Chinnery PF, Turnbull DM, Howell N (1997). Mammalian mitochondrial genetics: heredity, heteroplasmy and disease. Trends Genet.

[18] Amato P, Tachibana M, Sparman M, Mitalipov S (2014). Three-parent in vitro fertilization: gene replacement for the prevention of inherited mitochondrial diseases. Fertil Steril.

[19] Avise JC (2004). Molecular markers, natural history, and evolution.

[20] Lavrov DV, Pett W (2016). Animal mitochondrial DNA as we don’t know it: mt-genome organization and evolution in non-bilaterian lineages. Genome Biol Evol.

[21] Pett W, Ryan JF, Pang K, Mullikin JC, Martindale MQ, Baxevanis AD (2011). Extreme mitochondrial evolution in the ctenophore *Mnemiopsis leidyi*: insight from mtDNA and the nuclear genome. Mitochondrial DNA.

[22] Müller M, Mentel M, van Hellemond JJ, Henze K, Woehle C, Gould SB (2012). Biochemistry and evolution of anaerobic energy metabolism in eukaryotes. Microbiol Mol Biol Rev.

[23] van der Giezen M (2009). Hydrogenosomes and mitosomes: conservation and evolution of functions. J Eukaryot Microbiol.

[24] Watanabe KI, Bessho Y, Kawasaki M, Hori H (1999). Mitochondrial genes are found on minicircle DNA molecules in the mesozoan animal *Dicyema*. J Mol Biol.

[25] Armstrong MR, Blok VC, Phillips MS (2000). A multipartite mitochondrial genome in the potato cyst nematode *Globodera pallida*. Genetics.

[26] Gibson T, Blok VC, Phillips MS, Hong G, Kumarasinghe D, Riley IT (2007). The mitochondrial subgenomes of the nematode *Globodera pallida* are mosaics: evidence of recombination in an animal mitochondrial genome. J Mol Evol.

[27] Shao R, Kirkness EF, Barker SC (2009). The single mitochondrial chromosome typical of animals has evolved into 18 minichromosomes in the human body louse, *Pediculus humanus*. Genome Res.

[28] Cameron SL, Yoshizawa K, Mizukoshi A, Whiting MF, Johnson KP (2011). Mitochondrial genome deletions and minicircles are common in lice (Insecta: Phthiraptera). BMC Genomics.

[29] Wei DD, Shao R, Yuan ML, Dou W, Barker SC, Wang JJ (2012). The multipartite mitochondrial genome of *Liposcelis bostrychophila*: insights into the evolution of mitochondrial genomes in bilateral animals. PLoS ONE.

[30] Worth JR, Yokogawa M, Isagi Y (2014). Outcrossing rates and organelle inheritance estimated from two natural populations of the Japanese endemic conifer *Sciadopitys verticillata*. J Plant Res.

[31] Havey MJ (1997). Predominant paternal transmission of the mitochondrial genome in cucumber. J Hered.

[32] Zouros E (2013). Biparental inheritance through uniparental transmission: the doubly uniparental inheritance (DUI) of mitochondrial DNA. Evol Biol.

[33] Breton S, Beaupré HD, Stewart DT, Hoeh WR, Blier PU (2007). The unusual system of doubly uniparental inheritance of mtDNA: isn’t one enough?. Trends Genet.

[34] Sato M, Sato K (2013). Maternal inheritance of mitochondrial DNA by diverse mechanisms to eliminate paternal mitochondrial DNA. Biochim Biophys Acta.

[35] Sutovsky P, Moreno RD, Ramalho-Santos J, Dominko T, Simerly C, Schatten G (1999). Ubiquitin tag for sperm mitochondria. Nature.

[36] DeLuca SZ, O’Farrell PH (2012). Barriers to male transmission of mitochondrial DNA in sperm development. Dev Cell.

[37] Nishimura Y, Yoshinari T, Naruse K, Yamada T, Sumi K, Mitani H (2006). Active digestion of sperm mitochondrial DNA in single living sperm revealed by optical tweezers. Proc Natl Acad Sci USA.

[38] Sato M, Sato K (2011). Degradation of paternal mitochondria by fertilization-triggered autophagy in *C. elegans* embryos. Science.

[39] Al Rawi S, Louvet-Vallée S, Djeddi A, Sachse M, Culetto E, Hajjar C (2011). Postfertilization autophagy of sperm organelles prevents paternal mitochondrial DNA transmission. Science.

[40] Mishra P, Chan DC (2014). Mitochondrial dynamics and inheritance during cell division, development and disease. Nat Rev Mol Cell Biol.

[41] Greiner S, Sobanski J, Bock R (2015). Why are most organelle genomes transmitted maternally?. Bioessays.

[42] Hurst LD (1995). Selfish genetic elements and their role in evolution: the evolution of sex and some of what that entails. Philos Trans R Soc Lond B Biol Sci.

[43] Hastings IM (1992). Population genetic aspects of deleterious cytoplasmic genomes and their effect on the evolution of sexual reproduction. Genet Res.

[44] Williamson D (2002). The curious history of yeast mitochondrial DNA. Nat Rev Genet.

[45] Christie JR, Schaerf TM, Beekman M (2015). Selection against heteroplasmy explains the evolution of uniparental inheritance of mitochondria. PLoS Genet.

[46] Stewart JB, Chinnery PF (2015). The dynamics of mitochondrial DNA heteroplasmy: implications for human health and disease. Nat Rev Genet.

[47] Sharpley MS, Marciniak C, Eckel-Mahan K, McManus M, Crimi M, Waymire K (2012). Heteroplasmy of mouse mtDNA is genetically unstable and results in altered behavior and cognition. Cell.

[48] Ma H, Xu H, O’Farrell PH (2014). Transmission of mitochondrial mutations and action of purifying selection in *Drosophila*. Nat Genet.

[49] Magoulas A, Zouros E (1993). Restriction-site heteroplasmy in anchovy (*Engraulis encrasicolus*) indicates incidental biparental inheritance of mitochondrial DNA. Mol Biol Evol.

[50] Nunes MD, Dolezal M, Schlötterer C (2013). Extensive paternal mtDNA leakage in natural populations of *Drosophila melanogaster*. Mol Ecol.

[51] Gyllensten U, Wharton D, Josefsson A, Wilson AC (1991). Paternal inheritance of mitochondrial DNA in mice. Nature.

[52] Doublet V, Souty-Grosset C, Bouchon D, Cordaux R, Marcadé I (2008). A thirty million year-old inherited heteroplasmy. PLoS ONE.

[53] Radojičic JM, Krizmanić I, Kasapidis P, Zouros E (2015). Extensive mitochondrial heteroplasmy in hybrid water frog (*Pelophylax* spp.) populations from Southeast Europe. Ecol Evol.

[54] Payne BA, Wilson IJ, Yu-Wai-Man P, Coxhead J, Deehan D, Horvath R (2013). Universal heteroplasmy of human mitochondrial DNA. Hum Mol Genet.

[55] Schwartz M, Vissing J (2002). Paternal inheritance of mitochondrial DNA. N Engl J Med.

[56] Pyle A, Hudson G, Wilson IJ, Coxhead J, Smertenko T, Herbert M (2015). Extreme-depth re-sequencing of mitochondrial DNA finds no evidence of paternal transmission in humans. PLoS Genet.

[57] Rokas A, Ladoukakis E, Zouros E (2003). Animal mitochondrial DNA recombination revisited. Trends Ecol Evol.

[58] Kondo R, Satta Y, Matsuura ET, Ishiwa H, Takahata N, Chigusa SI (1990). Incomplete maternal transmission of mitochondrial DNA in *Drosophila*. Genetics.

[59] Dokianakis E, Ladoukakis ED (2014). Different degree of paternal mtDNA leakage between male and female progeny in interspecific *Drosophila* crosses. Ecol Evol.

[60] Birky CW (2001). The inheritance of genes in mitochondria and chloroplasts: laws, mechanisms, and models. Annu Rev Genet.

[61] Stern DB, Palmer JD (1984). Recombination sequences in plant mitochondrial genomes: diversity and homologies to known mitochondrial genes. Nucleic Acids Res.

[62] Taylor JW (1986). Fungal evolutionary biology and mitochondrial DNA. Exp Mycol.

[63] Wilson AC, Cann RL, Carr SM, George M, Gyllensten UB, Helm-Bychowski KM (1985). Mitochondrial DNA and two perspectives on evolutionary genetics. Biol J Linn Soc.

[64] Thyagarajan B, Padua RA, Campbell C (1996). Mammalian mitochondria possess homologous DNA recombination activity. J Biol Chem..

[65] Ladoukakis ED, Zouros E (2001). Direct evidence for homologous recombination in mussel (*Mytilus galloprovincialis*) mitochondrial DNA. Mol Biol Evol.

[66] Kraytsberg Y, Schwartz M, Brown TA, Ebralidse K, Kunz WS, Clayton DA (2004). Recombination of human mitochondrial DNA. Science.

[67] Ma H, O’Farrell PH (2015). Selections that isolate recombinant mitochondrial genomes in animals. Elife.

[68] Ladoukakis ED, Zouros E (2001). Recombination in animal mitochondrial DNA: evidence from published sequences. Mol Biol Evol.

[69] Piganeau G, Gardner M, Eyre-Walker A (2004). A broad survey of recombination in animal mitochondria. Mol Biol Evol.

[70] Tsaousis AD, Martin DP, Ladoukakis ED, Posada D, Zouros E (2005). Widespread recombination in published animal mtDNA sequences. Mol Biol Evol.

[71] Ciborowski KL, Consuegra S, de Leániz CG, Beaumont MA, Wang JL, Jordan WC (2007). Rare and fleeting: an example of interspecific recombination in animal mitochondrial DNA. Biol Lett.

[72] Edgar RC, Haas BJ, Clemente JC, Quince C, Knight R (2011). UCHIME improves sensitivity and speed of chimera detection. Bioinformatics.

[73] Hagström E, Freyer C, Battersby BJ, Stewart JB, Larsson NG (2014). No recombination of mtDNA after heteroplasmy for 50 generations in the mouse maternal germline. Nucleic Acids Res.

[74] Muller HJ (1964). The relation of recombination to mutational advance. Mutat Res.

[75] Felsenstein J (1974). The evolutionary advantage of recombination. Genetics.

[76] Martin WF, Garg S, Zimorski V (2015). Endosymbiotic theories for eukaryote origin. Philos Trans R Soc Lond B Biol Sci.

[77] Loewe L (2006). Quantifying the genomic decay paradox due to Muller’s ratchet in human mitochondrial DNA. Genet Res.

[78] Lynch M, Koskella B, Schaack S (2006). Mutation pressure and the evolution of organelle genomic architecture. Science.

[79] Neiman M, Taylor DR (2009). The causes of mutation accumulation in mitochondrial genomes. Proc Biol Sci.

[80] Gordo I, Charlesworth B (2000). The degeneration of asexual haploid populations and the speed of Muller’s ratchet. Genetics.

[81] Schierup MH, Hein J (2000). Consequences of recombination on traditional phylogenetic analysis. Genetics.

